# Effect of anatomical studies prior to medical school on medical school anatomy study success and residency choice

**DOI:** 10.1186/s12909-024-05642-5

**Published:** 2024-07-29

**Authors:** Brianna L. Walter, Rebecca L. Pratt

**Affiliations:** 1 Oakland University William Beaumont School of Medicine, Rochester, MI USA; 2 Department of Foundational Medical Studies, Oakland University William Beaumont School of Medicine, Rochester, MI USA

**Keywords:** Anatomy, Specialty choice, Academic success, Historically under-represented identity, Diversity

## Abstract

**Introduction:**

There is little research on how medical school matriculants’ experiences prior to medical school effects their choice of specialty or performance in medical school. This research attempts to fill that gap in research in regards to surgical and radiological fields.

**Methods:**

An anonymous survey was sent to fourth year medical students asking them their background in anatomy studies prior to medical school and their anatomy grades in medical school and interest in surgical and radiological fields. Students were also asked whether they identified with under-represented demographic groups in those specialties.

**Results:**

Prior anatomy experience did not significantly affect performance in anatomy courses or Step 1 and Step 2 scores. However, students who applied to surgical specialties had higher performance in anatomical courses and higher Step 1 scores compared to those who did not apply to surgical specialties. There was interestingly no difference in under-represented and not under-represented student application to these fields.

**Discussion:**

For shareholders interested in increasing medical student performance, or interest in specific specialties, more information needs to be gathered.

## Introduction

What pre-matriculation factors influence specialty choice? There is a paucity of research on the long-term effects of pre-matriculation factors, including specific coursework. This would be illuminating information for the many shareholders in the medical education process, including undergraduate and medical students, pre-medical advisors, medical school admissions committees, and residency directors seeking strategies to increase interest by historically under-represented groups in their specialty. The webpage of the American Medical Association (AMA) on choosing an undergraduate major includes a testimonial from a medical student who says, “some students have found a science-focused major gives them the foundation to… hit the ground running once they enter medical school” [1]. However, the AMA guide does not offer any advice on specific class work that may be helpful. Their closing message is that students should simply study what they are “passionate” about. The consensus from a majority of pre-medical students is that there is little room for self-discovery and development in undergraduate coursework. Instead, science-focused studies are an intense sequence of challenges and obstacles [2]. The lack of published data regarding undergraduate coursework and its effect on discipline success makes it harder for a prospective student to evaluate the long-term effects of their choice in undergraduate course work prior to matriculation at a medical school. There is a critical need to gather this information so that students can be informed upfront. Unfortunately, the default in course selection may be the courses which make it easier to attain the high GPA often required for a successful application.

The topic of interest for this work is whether anatomy coursework prior to medical school affects medical student academic success and residency choice. As anatomy is not tested on the Medical College Admissions Test (MCAT), students pursuing US-based medical schools which require MCAT for admission might not consider it a practical course to take prior to medical school. In 2002, Forester et al. found that completing anatomy courses prior to medical school had a positive impact on medical school anatomy and histology grades [3]. No statistically significant course performance difference was determined between those who had and those who had not had anatomy coursework previous to enrollment in medical school. However, the qualitative component of the paper supported perceived gains with a background in anatomy. “When asked at the completion of the course if they would recommend an anatomy course prior to professional school, 240 of the 250 students (96%) who completed the post course survey answered “yes”” [4]. Previous research has also shown that pre-matriculation modules provided a statistically significant increase in grades for students who accessed these modules. At one United States medical school, faculty developed an anatomy module which was sent to all accepted and waitlisted applicants eleven weeks prior to classes starting. It was found that students who utilized this resource had a statistically significant increase in grades. This increase was independent of academic ability or motivation [5]. Modules in biochemistry and physiology at a different United States medical school also provided statistically significant increase in grades, despite students who accessed the modules having the same baseline GPA and MCAT as those who did not access the modules [6]. However, these studies did not survey students about specialty choice – prior research on undergraduate interventions has not investigated the connection to medical professional’s career path beyond medical school.

A literature search of peer-reviewed materials demonstrates that little has been studied about the effectiveness of learning about a specialty in medical school and whether this influences student’s selection for residency. It was determined that students who learn about plastic surgery in the medical school curriculum are more likely to apply to plastic surgery residencies [7, 8]. In orthopedic surgery, female applicants showed a similar increased interest due to required curriculum exposure [9]. A similar effect was also found on students who took a curriculum with increased didactic time in diagnostic radiology [10].

There are several populations of interest when discussing groups that are historically under-represented in these fields. Women represent a minority in the field of surgery [11]. Additionally, they represent less than 20% of interventional radiology residents and 22% of diagnostic radiology residents [12]. Sexual and gender minorities, such as LGBT persons and those identifying with a non-binary gender, have been found to view surgery as a place where they will not be accepted. There is a proportional disinterest among sexual and gender minorities in pursuing these specialties [13]. Previous research has shown that in the 1980–2000’s, surgeons were disproportionately more likely to have a significantly negative view of gay and lesbian physicians and medical school applicants, along with stating intent not to refer to gay and lesbian colleagues [14, 15]. This finding was not as significant among radiologists. Sending out the same survey in 2018 showed that the average physician now has much less stigmatizing views of LGBT and HIV + medical professionals, and that surgery is now as accepting on average as other specialties [16]. Members of historically under-represented ethnic and/or racial identities remain under-represented in specialty and subspecialty care at large: according to AAMC, only about 30.6% of white physicians practice primary care, and 37–42% of physicians of other races practice primary care as opposed to specializing [12]. Members of these identities are also under-represented in surgery and radiology both in comparison to population and as a proportion of medical school graduates [17–20]. One exception in a surgical field is obstetrics, which has a more proportionate number of ethnic and gender minority residents and physicians [21].

There is some data about personality differences between surgeons and non-surgeons, such as surgeons having increased conscientiousness and increased interest in career prestige and remuneration [22]. These personality traits have been shown to predict some specialty choices even when assessed prior to medical school matriculation [23]. The findings of that study did not find statistical significance for predicting surgical or radiological specialties. Certain personality elements, such as empathy, aloofness, and self-confidence have also been found to have a statistically significant correlation with academic success as measured by class decile, number of publications, and additional degrees achieved in medical school in the United Kingdom [24]. However, there does not seem to be prior interventions utilizing this data in ways relevant to our interest in increasing representation in surgical and radiologic specialties.

Given the difficulty of being accepted into a medical school, the restricted number of seats in a year, and the looming physician shortage, it is in the best interest of students, medical school administration, and the general public that each matriculating student be provided with ample and effective opportunities to learn. The goal of this study was to investigate how pre-matriculation coursework affects the ability of students to learn and retain information on anatomy. Additionally, we looked at exposure to specific specialties prior to medical school and the effects they had on the selection process of graduating students from a historically under-represented background. The specialties of radiology and surgery were chosen because nationally there is disproportionate representation of students matching into these residencies. We hypothesized that early exposure to anatomy, the fundamental discipline that a surgeon and radiologist use in everyday practice, could be key to driving interest in these specialties.

## Methods

A retrospective model was used in which a survey was sent to fourth year medical students (MS4’s) at Oakland University William Beaumont School of Medicine (OUWB-SOM) in two consecutive years (Winter semesters 2021 and 2022 prior to the Match). A follow-up email was sent with a reminder to complete the survey in the hope of increasing responses. Additionally, other MD schools in the state of Michigan were contacted with the goal of sending the survey to their MS4 email listserves. However, this attempt was unsuccessful, as school administrators wished to lower the number of emails their students received. Thus only OUWB-SOM MS4 students were included in the final survey sample.

A primary concern was to protect the identity of respondents. Given the small number of MS4’s in one class, they were not asked to identify with a specific ethnic, gender, or sexual group; rather, they were presented with all the categories that are encompassed by the term ‘historically under-represented’ and asked a binary yes-no question on whether they identify with at least one category. While this made the data somewhat less granular for analysis, it did provide a higher level of protection for students who may be one of two or three in their class who represent a certain identity.

For the aim of investigating the effect of anatomy prior to medical school on medical school anatomy grades, the students were asked whether they took an anatomy course prior to medical school. As in previous research [4], an anatomy class will be defined as “any course with a primary focus on the physical structures of the body, human or vertebrate.” Similar to Stoddard’s approach, an element of the survey was directed at removing ‘interest in anatomy’ as a confounding variable [5]. The question was asked using a simple Likert scale to gauge interest in anatomy, and further questions were asked on level of education prior to medical school, whether students who did take anatomy took it for personal interest or to fulfill a graduation requirement, and whether students who did not take it simply did not have the opportunity to do so. Students were asked to self-report what percentile grade ranges they got in Anatomical Foundations of Clinical Practice (AFCP) 1 and 2 (these are the first-year anatomy courses at OUWB-SOM). Participants then self-reported their STEP 1 score and STEP 2 score, also within a percentile range to remove STEP performance as a potential confound for perceived competitiveness in applications. Percentiles were used for ease of data interpretation to standardize the measure of performance between cohorts.

To investigate the aim of whether students who took anatomy are more likely to be interested in a surgery or radiology career, students were asked to report interest in these fields using a Likert scale and whether they applied for a residency program in either of these two specialties. Lastly, students were given an opportunity to write a narrative about their interest/disinterest in these fields. The narrative portion was optional for completion of the study.

## Results

From the two class cohorts who received the survey (125 students/cohort), a total of 43 responded. Thirteen (30.2%) identified themselves with a historically under-represented group (i.e. ethnic/racial minority, gender and/or sexual minority) and thirty (69.8%) did not. There were 38 students with Bachelor’s degrees (88.4%), 4 students with Master’s degrees (9.3%), and 1 student with a doctorate level degree prior to medical school (2.3%). Anatomy experience prior to medical school was fairly evenly split, with 20 students having taken an anatomy course (46.5%) and 23 not having taken an anatomy course (53.5%). Of these 20 students with anatomy experience prior to medical school, 8 were required to take anatomy by their program (40%) and 12 took it because they wanted to (60%). Of the 23 students who did not take anatomy, 13 wanted to but could not afford the class (56.5%), 4 went to a school were these classes were not offered (17.4%), 3 were not required to take these classes for their major (13.0%), 1 did not take it because it was not necessary for their medical school prerequisites (4.3%), and 1 student was told by an advisor to wait until medical school (4.3%). Interest in anatomy as rated on a Likert scale 1–5 with 1 being no interest and 5 being a great deal of interest found a mean of 4.0 (SD 1.1) and a median of 4.0, representing overall high interest in anatomy as a subject.

An interest in surgery was rated on a Likert scale 1–5 with 1 being no interest and 5 being a great deal of interest found a mean of 2.7 (SD 1.8) and a median of 2.0. Fifteen students (34.9%) planned to apply to a residency program in general surgery or a surgical subspecialty. An interest in radiology was rated with a mean of 2.1 (SD 1.4) and median 2.0. Four students (9.3%) planned to apply to a residency program in radiology and thirty-nine students (90.7%) planned not to apply to such a program.

The effect of prior anatomy experience on medical school grades was assessed with Fisher’s Exact test, comparing prior anatomy experience vs. no prior experience with the categorical variable of grade percentile rank. There was no significant effect of prior experience on performance in AFCP 1 (*p* = 0.3961) or AFCP 2 (*p* = 0.6331). There was no significant effect on performance in STEP 1 (*p* = 0.3919) or STEP 2 CK (*p* = 0.6062).

To assess which variables had an association with applying to a surgical specialty, categorical variables were compared using a Fisher’s Exact test and continuous variables were compared using a 2-sample t-test. *P*-values less than 0.05 were considered statistically significant. Treating AFCP1 and AFCP2 as continuous variables yielded a statistically insignificant but functionally relevant (*p* = 0.10) result, with students that applied to a surgical specialty having higher performance in those courses than those that did not apply to a surgical specialty. For the STEP 1 exam, students who applied to surgery and surgical subspecialties scored higher than those who did not (*p* = 0.0008). When STEP 2 CK was assessed as a continuous variable, the effect was significant (*p* = 0.0030); this effect was not significant when assessed as a categorical variable (*p* = 0.1286). Interest in anatomy as a subject did not have a statistically significant effect on applying to a surgical residency (*p* = 0.18). Interest in surgery, however, had an effect on applying to a surgical residency (*p* < 0.0001). There was no association between application to a surgical specialty and anatomy experience prior to medical school (*p* = 1). Being from an under-represented population in surgery also did not have any statistically significant effect on applications to a surgical specialty (*p* = 1).

To assess which variables had an association with applying to radiology, categorical variables were compared using a Fisher’s Exact test and continuous variables were compared using a 2-sample t-test. *P*-values 0.05 were considered statistically significant. There was no statistically significant effect of AFCP 1 and 2 course grades on the likelihood of applying for a radiology residency (*p* = 1 and 0.45, respectively). For the STEP 1 exam, students who applied to radiology did not score significantly higher than those who did not (*p* = 0.078). The same was true for STEP 2 CK (*p* = 0.60). Interest in anatomy as a subject did not have a statistically significant effect on applying to a radiology residency program (*p* = 0.48). Interest in radiology, however, had an effect on applying to a radiology residency (*p* < 0.0001). There was no association between application to radiology and anatomy experience prior to medical school (*p* = 0.80). Being from an under-represented population in radiology also did not have a statistically significant effect on applications to a radiology residency (*p* = 0.30).

Logistic regression was used to examine the effect of being from an under-represented population had on the relationship between prior anatomy experience and applying to a surgical specialty. Firth’s correction for small sample sizes (Firth’s Penalized Likelihood Logistic Regression) was used to account for the low sample size in groups. The outcome was applying to a surgical specialty and the independent factors were having anatomy experience prior to medical school, being from an under-represented population, and the interaction between these two independent factors. The *p*-value for the interaction was 0.50, meaning that there was not a statistically significant interaction between these factors in the outcome of surgery subspecialty applications.

In the survey section offered for students to further expand on their answers, only 11 responded, with the majority of the responses being one- or two-word comments indicating specialty of choice. Two students explained why they were not interested in surgery and/or radiology, citing personality clash, lifestyle, perceived lack of continuity of care, and (for radiology) perceived lack of patient interaction as key factors in their decision.

## Discussion

We would like to address first and foremost a limitation of our study that impacts our data. We recognize that this study was limited by a small sample size; total *n* = 43. Multiple interventions were made to increase this sample size, including sending the survey to two consecutive cohorts of fourth year students (125 students/cohort), sending reminder emails as well as contacting other state medical schools in an attempt to expand the population size. Future research on influential factors on anatomy success and interests in anatomy-driven residency specialties should aim to include a larger sample size with survey incentives and or multiple medical school cohorts. We do caution that by opening the survey to multiple medical schools more external variables will be introduced. For example, but not limited to, differences in curricula, differing recruitment tools utilized by schools to open opportunities for historically under-represented students, tuition waivers, residency seats offered, and undergraduate schools that have a medical school associated with their campus. These differences could confound the data; however, they would also make it more generalizable to the broad population of pre-medical and medical students.

One goal of this study was to determine if exposure to anatomy coursework prior to medical school matriculation would have an effect on medical school grade performance and on residency interest. It is crucial that we identify determinants of success for all medical students and especially under-represented minority students. A better understanding of these factors would help inform premedical advising, prerequisite coursework for matriculation to American medical schools, and interventions for stakeholders with a strong desire to increase diversity in a historically homogeneous application pool. The statistically significant findings of this study are limited to what has already been published - students who choose to apply to surgical subspecialties generally have a higher STEP score average than students applying to non-surgical specialties [25]. With that stated, we do feel that this study clearly highlights the effect of other, lesser-known determinants of student success in medical school and in residency. More important for consideration is how these factors apply to members of historically under-represented identities.

A notable finding of this study was that among this small sample there was no statistically significant effect on the under-represented population of fourth year OUWB-SOM students’ status on their likelihood of applying to a surgical or radiology residency. The knowledge that these self-identified students feel that these residencies are a possibility can be considered a positive point. We recognize that these populations are considered historically under-represented for a reason. Therefore, these findings could indicate that the students who responded to the survey are not representative of the national pool of under-represented minority graduates applying to residency. Repeating this study with a larger response group could be beneficial to illuminating a clearer result. Additionally, repeating similar research in a population distributed over a larger geographic area could better control for institutional cultural differences that could be a contributing factor to these findings.

Prior research has shown that a feeling of not belonging or feeling welcome within a field of study can negatively impact the rate of application by under-represented students [13]. In conceiving this study, we had hypothesized that early exposure to anatomy coursework prior to matriculation might increase grades in anatomy and also reduce this feeling of not belonging in specialties with a heavy focus on anatomy in practice. However, early exposure to anatomy courses was not shown by this study to improve grades or to increase the rate of applications to surgical or radiological residency programs. The only statistically significant factors in increasing applications to surgery and surgical-subspecialties were STEP 1 and STEP 2 CK scores and a prior interest in surgery as a specialty. Neither of these were affected by having taken an anatomy class prior to medical school. Additional interventions, such as the availability of diversity among faculty mentors or early exposure to these specialties could be considered in the future.

Personality traits have been shown to affect some of our outcomes of interest, such as specialty choice and medical school grades [22–24]. However, the intersections of those factors with elements effecting under-represented minority interest in these specialties is not yet well understood. Future research could be done for interventions in recruiting students based on personality traits for specialties of interest or specialties facing shortages of providers.

Our data does demonstrate that not only is there a strong interest in participating in an anatomy course prior to matriculation, but that a majority of medical-school bound students actually have this opportunity. Interest in anatomy appeared to be high, with the average student rating their interest a 4 out of 5 on Likert scale. Furthermore, of the 53.5% of students who had not taken anatomy prior to medical school, only 17% stated that this was because no course was offered at their school, indicating that these courses are accessible for interested students.

Interestingly, while two students reported that they had not taken anatomy due to being told not to take it by an advisor, none of those who took it reported that they had been advised to do so. These responses highlight a subject that requires further investigation. How are pre-health advisors educated on what makes a successful applicant to medical schools? It seems that anatomy courses are not given a strong consideration in the repertoire of pre-medical coursework, as far as undergraduate advising is concerned. Our data suggest that there is a disconnect between what undergraduate students desire, what is offered to them in undergraduate course catalogs, what they are advised to study, and what subjects will be most helpful in medical school.

## Conclusion

Analysis of first-year medical school anatomy and STEP 1 and 2 CK scores showed that students who took anatomy prior to medical school had no improved performance over those who did not take anatomy prior to medical school. Of the measures taken in this study, only STEP 1 and 2 CK scores had a statistically significant effect on the choice of residency specialty among all students. Based on this study’s sample size, an anatomy course requirement or recommendation prior to medical school may or may not be an efficacious intervention for raising medical school gross anatomy grades, STEP scores, prior student interest in surgery and radiology, or historically under-represented persons’ interest in these fields. Previous literature has shown a positive correlation with residency specialty introductions during medical influencing residency program selection. For stakeholders desiring to increase residents from historically under-represented backgrounds in radiology and surgery, it will be important to increase the number of respondents. We do not believe that the factors we chose to investigate in our hypothesis should be abandoned until a larger sample is collected and analyzed.

In summary, this research fills a gap in the literature by exploring the effect of pre-medical school anatomy education on medical student academic success and residency choice. While this study did not find significant associations between prior anatomy experience and the variables examined, it does emphasize the importance of further investigation in this field. The limitations of the study have been discussed, and future research directions suggested.

## Data Availability

The datasets used and/or analysed during the current study are available from the corresponding author on reasonable request.
